# Effects of Variability in Blood Pressure, Glucose, and Cholesterol Concentrations, and Body Mass Index on End-Stage Renal Disease in the General Population of Korea

**DOI:** 10.3390/jcm8050755

**Published:** 2019-05-27

**Authors:** Mee Kyoung Kim, Kyungdo Han, Hun-Sung Kim, Yong-Moon Park, Hyuk-Sang Kwon, Kun-Ho Yoon, Seung-Hwan Lee

**Affiliations:** 1 Division of Endocrinology and Metabolism, Department of Internal Medicine, Yeouido St. Mary’s Hospital, College of Medicine, The Catholic University of Korea, Seoul 07345, Korea; makung@catholic.ac.kr (M.K.K.); drkwon@catholic.ac.kr (H.-S.K.); 2 Department of Medical Statistics, College of Medicine, The Catholic University of Korea, Seoul 06591, Korea; hkd917@naver.com; 3 Division of Endocrinology and Metabolism, Department of Internal Medicine, Seoul St. Mary’s Hospital, College of Medicine, The Catholic University of Korea, Seoul 06591, Korea; 01cadiz@hanmail.net (H.-S.K.); yoonk@catholic.ac.kr (K.-H.Y.); 4 Department of Medical Informatics, College of Medicine, The Catholic University of Korea, Seoul 06591, Korea; 5 Epidemiology Branch, National Institute of Environmental Health Sciences, National Institutes of Health, Research Triangle Park, NC 27709, USA; markparkjecos@gmail.com

**Keywords:** ESRD, variability, metabolic parameters

## Abstract

Aim: Metabolic parameters, such as blood pressure, glucose, lipid levels, and body weight, can interact with each other, and this clustering of metabolic risk factors is related to the progression to end-stage renal disease (ESRD). The effect of variability in metabolic parameters on the risk of ESRD has not been studied previously. Methods: Using nationally representative data from the Korean National Health Insurance System, 8,199,135 participants who had undergone three or more health examinations between 2005 and 2012 were included in this analysis. Intraindividual variability in systolic blood pressure (SBP), fasting blood glucose (FBG), total cholesterol (TC), and body mass index (BMI) was assessed by examining the coefficient of variation, variability independent of the mean, and average real variability. High variability was defined as the highest quartile of variability and low variability was defined as the lower three quartiles of variability. Results: Over a median (5–95%) of 7.1 (6.5–7.5) years of follow-up after the variability assessment period, 13,600 (1.7/1000 person-years) participants developed ESRD. For each metabolic parameter, an incrementally higher risk of ESRD was observed for higher variability quartiles compared with the lowest quartile. The risk of ESRD was 46% higher in the highest quartile of SBP variability, 47% higher in the highest quartile of FBG variability, 56% higher in the highest quartile of BMI variability, and 108% higher in the highest quartile of TC variability. Compared with the group with low variability for all four parameters, the group with high variability for all four parameters had a significantly higher risk for incident ESRD (hazard ratio (HR) 4.12; 95% CI 3.72–4.57). Conclusions: Variability in each metabolic parameter was an independent predictor of the development of ESRD among the general population. There was a composite effect of the variability in additional metabolic parameters on the risk of ESRD.

## 1. Introduction

End-stage renal disease (ESRD) has emerged as one of the most important public health issues. Old age, diabetes mellitus (DM), hypertension (HTN), and hyperuricemia have been identified as traditional independent risk factors for ESRD [1]. Despite the recent advances in the management of ESRD, mortality rates in patients with ESRD remain high [1,2]. Therefore, it is important to prevent the progression of ESRD through the early detection of people at high risk and treatment of modifiable factors.

Recent interest in the intraindividual variability in various metabolic parameters has led to the recognition of their role as a risk factor for health-related outcomes [3,4,5,6,7,8]. Among people with type 2 DM, glucose variability is an independent predictor of ESRD after adjusting for other conventional risk factors, including mean glucose level [9]. Moreover, a growing body of evidence suggests that blood pressure (BP) variability portends worse outcomes in both the general population and in people with kidney disease. Previous studies have identified BP variability as a risk factor for the progression of chronic kidney disease (CKD), strokes, and mortality [5]. BP variability is also a compelling risk factor among patients undergoing hemodialysis [10]. The absence of nocturnal dipping, BP increase over the course of dialysis, and visit-to-visit prehemodialysis BP variability have all been linked to increased mortality [10]. Greater visit-to-visit BP variability has significant prognostic value and points to some potentially modifiable practice patterns, including antihypertensive agent selection, to achieve consistent BP levels.

We previously reported that cholesterol variability is an independent risk factor for the development of ESRD in the general population [11]. Metabolic parameters such as BP, glucose, lipid levels, and body weight can interact with each other, and the clustering of metabolic risk factors is related to the progression of ESRD [12]. However, the full effect of the variability in metabolic parameters on the risk of ESRD has not been studied previously and remains to be better understood. To examine the prognostic significance of increased variability in metabolic parameters (glucose and cholesterol concentrations, BP, and body mass index (BMI)) on the development of ESRD, we conducted a large population-based study involving more than 8 million Koreans who had at least three measurements of all parameters.

## 2. Methods

### 2.1. Study Participants

The Korean National Health Insurance System (NHIS) comprises a complete set of health information pertaining to about 50 million Koreans. This study used the entire NHIS–National Health Examinee database, which is a database of all individuals who underwent National Health Examinations. The data include qualification data, medical services claim data, and pharmacy claim data. The NHIS is a single-payer organization that is managed by the government, to which all residents in Korea subscribe. Enrollees in the National Health Insurance Corporation are recommended to undergo standardized medical examinations every 2 years. Details of the database were previously described [3,13,14]. In our study, we included people who had undergone a health examination in 2009 or 2012 (index year) and three or more health examinations between 1 January 2005 and 31 December 2012. Of 17,374,695 participants (age ≥ 20 years) with health examination data in the index year, 8,211,563 underwent three or more health examinations during this period. We excluded 6391 people with missing data for at least one variable. Those having ESRD (n = 6037) before the index year were also excluded. Ultimately, the study population comprised 8,199,135 people (Figures S1 and S2). This study was approved by the Institutional Review Board of The Catholic University of Korea (No. KC18EESI0429). Deidentified information was used for analyses, and informed consent was not required.

### 2.2. Measurements and Definitions

BMI was calculated as weight in kilograms, divided by the square of height in meters. Information on current smoking and alcohol consumption (heavy alcohol consumption defined as ≥30 g/day) was obtained by questionnaire. Regular exercise was defined as performing more than 30 min of moderate physical activity at least five times per week or more than 20 min of strenuous physical activity at least three times per week. Income level was dichotomized at the lowest 25%. Blood samples for the measurement of serum glucose, creatinine (Cr), and lipid levels were drawn after an overnight fast. The estimated glomerular filtration rate (eGFR) was calculated using the abbreviated Modification of Diet in Renal Disease formula: 175 × serum Cr (mg/dL)^−1.154^ × Age (year)^−0.203^ × 0.742 (if female) [15]. Low eGFR was defined as an eGFR < 60 mL/min/1.73 m^2^, according to the U.S. National Kidney Foundation guidelines [16]. Urine protein was measured semiquantitatively with a urine dipstick tested on fresh, midstream urine samples and was reported as the following six grades: Absent, trace (±), 1+, 2+, 3+, and 4+, which correspond to protein levels of undetectable, 10, 30, 100, 300, and 1000 mg/dL, respectively. Proteinuria was defined as a grade of 1+ or greater. Hospitals in which these health examinations were performed were certified by the NHIS and subjected to regular quality control.

The presence of DM was defined according to the following criteria: (1) At least one claim per year under International Classification of Disease, 10th Revision (ICD-10) codes E10–14 and at least one claim per year for the prescription of antidiabetic medication or (2) a fasting blood glucose (FBG) concentration ≥ 126 mg/dL [17]. The presence of HTN was defined according to the presence of at least one claim per year under ICD-10 codes I10 or I11 and at least one claim per year for the prescription of an antihypertensive agent or systolic/diastolic BP ≥ 140/90 mmHg. The presence of dyslipidemia was defined according to the presence of at least one claim per year under ICD-10 code E78 and at least one claim per year for the prescription of lipid-lowering agent or total cholesterol (TC) concentration ≥ 240 mg/dL.

### 2.3. Definition of Variability and Scoring

We used three metrics to describe the variability in metabolic parameters: Coefficient of variation (CV), variability independent of the mean (VIM), and average real variability (ARV). Variability indices must adequately reflect fluctuations in parameters without overinfluence from ambient parameters levels, such that the independent effects of each can be distinguished. The CV measures variability better than standard deviation (SD): The latter correlates with absolute parameter values, and the former normalizes it [18,19]. VIM was calculated as 100 × SD/mean^β^, where β is the regression coefficient using the natural logarithm of SD divided by the natural logarithm of the mean [20]. ARV is the average of the absolute differences between consecutive values [19].

The number of measurements per participant ranged as follows: Three measurements (*n* = 5,441,487 or 66%), four measurements (*n* = 1,296,955 or 16%), and five measurements (*n* = 1,460,693 or 18%). High variability was defined as the highest quartile (Q4) of variability and low variability as the lower three quartiles (Q1–3) of variability. The participants were classified further according to the number of high-variability metabolic parameters (FBG, TC, SBP, and BMI) using a score range from 0 to 4. In this classification, a score of 0 indicated no high-variability parameter and the scores 1–4 indicated the number of high-variability parameters of the four total parameters.

To consolidate our findings, we analyzed the above relationships using other definitions of variability. We defined the variability score as 0 points to Q1 (lowest quartile of variability), 1 point to Q2, 2 points to Q3, and 3 points to Q4 (highest quartile of variability) for each of the four parameters (glucose variability, cholesterol variability, SBP variability, and BMI variability). We then summed these to give a variability score ranging from 0 to 12 points.

### 2.4. Study Outcomes and Follow-Up

The study population was followed from baseline to the date of ESRD diagnosis or until 31 December 2016, whichever came first. The primary end point was incident ESRD, which was defined using the combination of ICD-10 code (N18–19, Z49, Z94.0, Z99.2) and initiation of renal replacement therapy and/or kidney transplantation (KT) during hospitalization. All medical care expenses for dialysis are reimbursed through the Korean Health Insurance Review and Assessment Service database. These patients are also registered as special medical aid beneficiaries. Therefore, we were able to identify every ESRD patient in the entire South Korean population and to analyze the data for all ESRD patients who had started dialysis. Codes for treatment or medical expense claims included R3280 for KT, O7011–O7020 or V001 for hemodialysis, and O7071–O7075 or V003 for peritoneal dialysis. We excluded individuals without previous chronic kidney disease who had a KT or dialysis code on the same date as an acute renal failure code. Participants on continuous renal replacement therapy or acute peritoneal dialysis were also excluded.

### 2.5. Statistical Analysis

Baseline characteristics are presented as the mean ± SD or n (%). The participants were classified into quartiles according to the measures of variability in metabolic parameters (FBG, SBP, TC, and BMI). The incidence rate of ESRD was evaluated for each of the quartiles. The incidence rate of ESRD was calculated by dividing the number of incident cases by the total follow-up duration (person-years). A Cox proportional-hazards regression analysis was performed to evaluate the risk of ESRD in the group with the highest quartile of variability versus the lowest quartile.

Participants were classified into five groups according to the number of high-variability metabolic parameters. The cumulative incidence of ESRD, according to the number of parameters with high variability, is presented using the Kaplan–eier curves, and the log-rank test was performed to analyze differences between groups. The hazard ratios (HRs) and 95% confidence intervals (CIs) for ESRD for the variability scores were analyzed using the Cox proportional-hazards model. The proportional-hazards assumption was evaluated using the Schoenfeld residuals test with the logarithm of the cumulative hazards function based on the Kaplan–Meier estimates for quartile groups of variability or groups based on the number of parameters with high variability. There was no significant departure from proportionality in hazards over time. The multivariable-adjusted proportional-hazards models were applied as follows: Model 1 was adjusted for age, sex, smoking, alcohol intake, regular exercise, and income status; model 2 was adjusted further for baseline FBG, SBP, TC, and BMI; and model 3 was adjusted further for baseline eGFR and the presence of proteinuria.

A sensitivity analysis was performed to exclude participants with end points occurring within 2 years of the follow-up to account for the possibility of reverse causation. A total of 3,013,396 people had DM, HTN, or dyslipidemia and had already received medical treatment before the index year. Because these treatments and drug compliance could affect measurement variability, we performed further analysis after excluding people with DM, HTN, or dyslipidemia.

The number of measurements can influence the variability. To overcome this limitation, we performed sensitivity analyses by including participants with five measurements (yearly measurements of metabolic parameters; 18% of the total study population). The potential effect modification by age, sex, BMI categories, DM, HTN, and low eGFR (<60 mL/min/1.73 m^2^) was evaluated using stratified analysis and interaction testing using a likelihood ratio test. Statistical analyses were performed using the SAS version 9.4 (SAS Institute Inc., Cary, NC, USA), and a *p* value of <0.05 was considered to indicate significance.

## 3. Results

### 3.1. Baseline Characteristics of the Study Population

The characteristics of the participants grouped according to the number of high-variability metabolic parameters are listed in Table 1. Participants with more high-variability parameters were older, more likely to be female, and had a higher prevalence of comorbid conditions (DM, HTN, dyslipidemia). The highest baseline FBG and triglyceride levels, and lower physical activity and income status were observed in participants with four high-variability parameters. The percentage of participants with an eGFR <60 mL/min/1.73 m^2^ or proteinuria increased gradually in those with more high-variability parameters. The VIM of each parameter increased gradually with the number of high-variability parameters. *p*-values for the trend were <0.0001 for all variables because of the large size of the study population.

The baseline CV of each parameter was significantly higher in participants with incident ESRD than in those without ESRD (Table 2). In participants with incident ESRD, the baseline FBG and triglyceride levels and systolic and diastolic BP were higher, and baseline high-density lipoprotein (HDL) cholesterol level and eGFR were lower. The prevalence of comorbid conditions, including DM, HTN, and dyslipidemia, was significantly higher in those with incident ESRD.

### 3.2. Risk of ESRD According to the Variability for Each Parameter

Over a median (5–95%) of 7.1 (6.5–7.5) years of follow-up after the variability assessment period, 13,600 participants (0.17%; 1.7/1000 person-years) developed ESRD. For each metabolic parameter, an incrementally higher risk of ESRD was observed for the higher variability quartiles compared with the lowest quartile group (Table 3). For the highest quartile of FBG variability compared with the lowest quartile, the risk of ESRD increased by 47%. For the highest quartile of SBP variability compared with the lowest quartile, the risk of ESRD increased by 46%. For the highest quartile of BMI variability compared with the lowest quartile, the risk of ESRD increased by 56% (HR 1.56; 95% CI 1.49–1.64). In particular, the highest quartile of TC variability was associated with a twofold increase in risk of developing ESRD (HR 2.08; 95% CI 1.97–2.18).

The association between variability for each parameter and ESRD was significant after adjusting for baseline FBG, TC, SBP, BMI, eGFR, and proteinuria.

### 3.3. Risk of ESRD According to the Number of High-Variability Parameters

**Figure 1:**
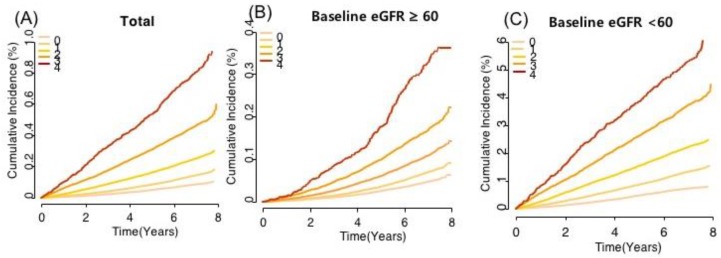
Kaplan–Meier estimates of cumulative incidence of end-stage renal disease (ESRD) according to the number of high-variability metabolic parameters: Total population (**A**), population with baseline estimated glomerular filtration rate (eGFR) ≥60 mL/min/1.73 m^2^ (**B**), and population with baseline eGFR <60 mL/min/1.73 m^2^ (**C**). High variability was defined as the highest quartile (Q4) of variability independent of the mean (VIM). Unadjusted Kaplan–Meier curves are presented because of the large sample size and relatively balanced distribution of baseline covariates.

**Figure 2:**
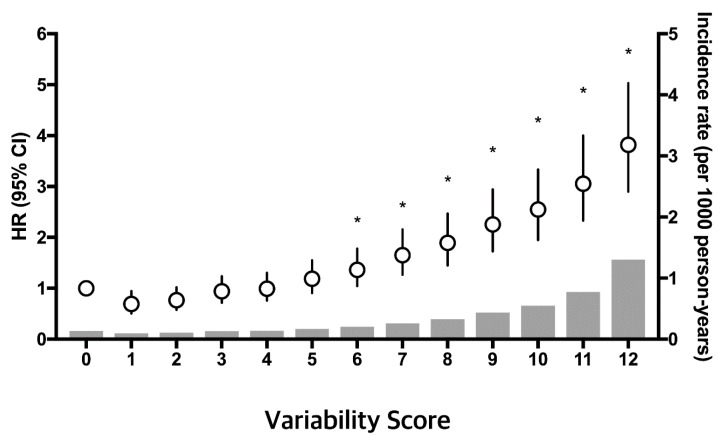
Incidence rates, hazard ratios, and 95% confidence intervals of end-stage renal disease (ESRD), according to variability score (0–12): 0 points was assigned to Q1 (lowest quartile of variability), 1 point to Q2, 2 points to Q3, and 3 points to Q4 (highest quartile of variability) for each of the four parameters (fasting blood glucose, total cholesterol, systolic blood pressure, and body mass index). The incidence rates of ESRD are represented by gray bars. The hazard ratio and 95% confidence intervals are shown as circles and solid lines, respectively. The analysis was adjusted for age, sex, alcohol intake, smoking, regular exercise, income, fasting blood glucose level, total cholesterol, systolic blood pressure, body mass index, estimated glomerular filtration rate, and proteinuria. * *p* < 0.05.

The number of high-variability parameters was linearly related to the incidence of ESRD (Table 4, Figure 1). The incidence of ESRD increased progressively with an increasing number of high-variability parameters in both participants with a baseline eGFR ≥60 mL/min/1.73 m^2^ and eGFR <60 mL/min/1.73 m^2^ (Figure 1). After adjusting for possible confounding factors, the HR values (95% CI) of ESRD were 1.54 (1.47–1.62) for one parameter, 2.25 (2.14–2.37) for two parameters, 3.17 (2.99–3.37) for three parameters, and 4.12 (3.72–4.57) for four parameters compared with participants with no high-variability parameters, measured as VIM. Within the variability score range of 0 to 12 points, multivariable-adjusted HRs for ESRD increased continuously and linearly with an increasing variability score (Figure 2).

### 3.4. Sensitivity Analysis

A total of 3,013,396 people had DM, HTN, or dyslipidemia and had already received medical treatment before the index year. Because these treatments and drug compliances can affect measurement variability, we analyzed the data further after excluding people with DM, HTN, or dyslipidemia (*n* = 5,185,739). Similar to the original analysis, an incrementally higher incidence rate and HR (95% CI) of ESRD was noted with an increasing number of high-variability parameters (Table 5), although the HRs were slightly attenuated.

Excluding participants with end points that occurred within 2 years of the follow-up produced incrementally higher incidence rates and HRs (95% CI) for ESRD with an increasing number of high-variability parameters (Table S1). Analysis confined to participants with yearly measurements of metabolic parameters also revealed similar results with higher HRs for ESRD compared with the original analysis (Table S2). The group with low variability for all four parameters among those who underwent annual health examinations had a very low incidence rate of ESRD (Table S2).

The combined effects of the variability in SBP and FBG, SBP and TC, and FBG and TC on the risk of ESRD was analyzed (Table S3). Compared with the group with low variability for two parameters, the group with high variability for one or two parameters had a significantly higher risk for incident ESRD. The HR values (95% CI) of ESRD were 1.87 (1.79–1.96) for the group with low variability of SBP and high variability of TC, 1.45 (1.38–1.52) for the group with high variability of SBP and low variability of TC, and 2.52 (2.40–2.65) for the group with high variability of SBP and high variability of TC compared with participants with no high-variability parameters.

### 3.5. Subgroup Analyses

**Figure 3:**
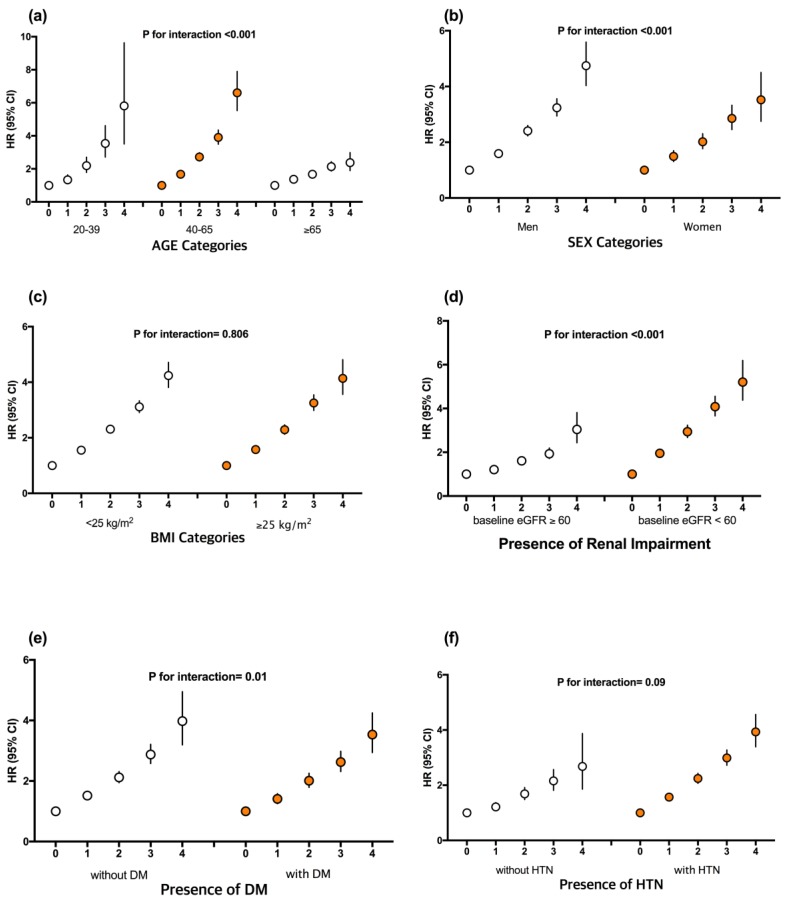
Hazard ratios and 95% confidence intervals of end-stage renal disease (ESRD) according to number of high-variability metabolic parameters. Subgroup analyses were stratified by age (**a**), sex (**b**), BMI category (**c**), presence of renal impairment (**d**), diabetes mellitus (**e**), and hypertension (**f**). The analysis was adjusted for age, sex, alcohol intake, smoking, regular exercise, income, fasting blood glucose level, total cholesterol, systolic blood pressure, body mass index, estimated glomerular filtration rate, and proteinuria.

A stratified analysis by age, sex, BMI category, impaired renal function (baseline eGFR ≥60 or eGFR <60 mL/min/1.73 m^2^), DM, and HTN was then conducted. A significant association between the number of parameters with high variability and the risk of ESRD was observed in all subgroups (Figure 3). Higher adjusted HRs for ESRD were observed in the subgroup that was younger and middle-aged (<65 years), male, eGFR <60 mL/min/1.73 m^2^, and without DM (*p* for interaction <0.05).

## 4. Discussion

Our results show that variability in each metabolic parameter is associated with an increased risk for ESRD among the general population. Of the metabolic parameters examined, lipid variability had the highest HR for ESRD. There was an additive effect of variability in metabolic parameters on the risk of ESRD. The risk of ESRD increased by more than fourfold in the group with high variability in all four parameters compared with that with low variability in all four parameters.

Maintaining metabolic parameters within a narrow range is mandatory. For example, the target for glycemia in healthy people is between 70 and 140 mg/dL. A longer time with blood glucose in this range is associated with increased survival in nondiabetic critically ill adults [21]. Over the long term, visit-to-visit glycemic variability predicts microvascular complications, including deterioration in renal function and macrovascular complications in patients with type 2 DM [22]. In one study, the highest quartile of the CV for hemoglobin A1c (HbA1c) levels was associated with a 1.6-fold higher risk for diabetic nephropathy compared with the lowest quartile, and the highest quartile of the CV for FBG was associated with a 4.8-fold higher risk for diabetic nephropathy compared with the lowest quartile [23].

In people with DM, wide variation in the CVs of FBG and HbA1c levels may reflect a more complicated clinical course, suboptimal medication, and poor self-management, or oxidative stress caused by acute variation in glucose levels. The FBG level captures acute fluctuations in glucose level caused by irregular eating or lifestyle factors, which are not easily detected by the HbA1c level. The CV of the FBG level is a new measure of glucose variation that can capture the association between oscillating plasma glucose levels and diabetic nephropathy [23]. Cell growth, collagen synthesis, and cytokine secretion in cultured human tubulointerstitial cells were increased after intermittent exposure to high glucose concentration compared with constant exposure to high glucose concentration [24]. In our study, FBG variability was associated with an increased risk of ESRD among the general population.

Increased SBP variability is more common in CKD patients and worsens with advancing CKD stages [25]. Increased BP variability in CKD may result from various pathophysiological mechanisms. The sympathetic nervous system is the final pathway for the regulation of BP circadian patterns by altering alpha-adrenergic activity, vascular tone, and catecholamine levels. Fluctuations in the activation of the renin-angiotensin-aldosterone system may lead to increased BP variability [25]. It was recently reported that visit-to-visit diastolic BP variability independently predicted cardiovascular outcomes as well as hypotension, syncope, and acute kidney injury in patients with CKD enrolled in the Systolic Blood Pressure Intervention Trial [26]. Greater BP variability may produce vascular damage because of the inability of certain vascular beds to maintain autoregulation over wider ranges of BP beyond a point, and the greater BP variability may contribute to adverse events [5]. The results of the present study suggest that BP variability is an independent risk factor for the development of ESRD, regardless of the presence of CKD. Our results also suggest that visit-to-visit variability in BP should be considered a new therapeutic target for renal protection, independent of the mean BP.

A higher risk for mortality was noted in participants who lost or gained weight compared with those with stable weight before dialysis initiation. Anorexia and weight loss accompany CKD progression [27,28]. Weight loss begins when serum Cr concentration is in the range of 1.5 to 2.0 mg/dL and earlier than may be expected [27]. An increase in body weight of ≥0.75 kg/year predicts incident CKD [29]. Excessive adiposity may be associated with renal injury. As with obesity-induced DM and HTN, the pathophysiology of obesity-related kidney disease may function through more subtle mechanisms, including a variety of hormonal and cytokine influences. Although BMI includes both adiposity and muscle mass, it correlates highly with adiposity, and relatively small changes in weight can significantly affect body fat level and cardiovascular or metabolic risk. Unfavorable health effects of weight fluctuation have been attributed to an increase in body fat mass, a decrease in resting energy expenditure, and an increase in abdominal fat [30]. In our study, variability in BMI, as reflected in weight fluctuation, was also an independent predictor of the development of ESRD among the general population.

We previously reported that increasing TC variability was associated with an increasing incidence of ESRD among the general population [11]. A growing body of evidence suggests that dyslipidemia may be a risk factor for the development of nephropathy in people with DM. People with a high triglyceride level are at greater risk of progression of diabetic nephropathy [31]. Higher high density lipoprotein-cholesterol (HDL-C) variability also significantly predicts the progression of diabetic nephropathy [32]. Either HDL deficiency or functional impairment of HDL-cholesterol may hamper the reverse cholesterol transport process and play an important role in glomerulosclerosis and tubulointerstitial damage [33]. Lipid variability may be an early marker of impaired renal function and may influence kidney function.

Our data clearly demonstrate that the variability in various metabolic parameters interacts in determining the final outcome, particularly in the development of ESRD. Increased variability might be a marker of an unhealthy status related to multimorbidity, poor quality of life, lack of social support, or frequent infectious complications. Increased variability might also be an indicator of a lack of compliance with healthy lifestyle behaviors. In this regard, we note that the participants in our study with higher variability at baseline were less physically active, had a lower income status, and had a greater prevalence of comorbidities than those with lower variability. However, similar results were obtained after excluding participants with DM, HTN, or dyslipidemia to exclude the potential influences of drug compliance or the disease itself. The association between ESRD and high variability in metabolic parameters cannot be explained only by poor drug compliance or comorbidities.

This study did have some limitations. First, excluding participants with fewer than three health examinations might have been a source of selection bias. Even though all Korean residents are entitled to biennial medical evaluation, less than half actually receive it because it is voluntary rather than obligatory. It is possible that those included in the present study may have maintained a healthier lifestyle and are therefore at lower risk. Second, we could not obtain further information about the specific causes of ESRD. Third, although we found a strong relationship between high variability in metabolic parameters and ESRD, we have insufficient evidence for a causal relationship. To minimize the possible effects of reverse causality, we excluded those with previous ESRD. The sensitivity analysis that excluded people with outcomes occurring in the first two years of follow-up also revealed similar results. High variability in metabolic parameters was associated with a higher risk of ESRD development in people without CKD.

## 5. Conclusions

The strengths of our study include its large sample size, inclusion of multiple important clinical and biochemical characteristics, and selection of a statistically robust variability metric. Whereas other studies focused mostly on diseased patients, we focused on the general population and not specific subgroups. We also analyzed the data after excluding participants with DM, HTN, or dyslipidemia to exclude the potential influences of medication compliance or the disease itself. Because the prevalence of ESRD is low, few studies have examined the association between metabolic variability and the development of ESRD. Larger studies over a longer time are needed to provide a more definitive answer about whether metabolic variability is a risk factor for ESRD. Our results add evidence that high variability in FBG and TC concentrations, SBP, and BMI is associated with a higher risk for ESRD development among the general population. Further research is required to elucidate the linkage mechanisms and to confirm whether metabolic variability is a valuable therapeutic target for identifying at-risk patients.

## Figures and Tables

**Table 1:** Baseline characteristics of subjects by the number of high variability in the metabolic parameters (fasting blood glucose, total cholesterol levels, systolic blood pressures, and body weight).

	0 (*n* = 2,782,077)	1 (*n* = 3,209,154)	2 (*n* = 1,678,400)	3 (*n* = 470,610)	4 (*n* = 58,894)
Age (years)	47.0 ± 12.6	48.1 ± 13.7	49.6 ± 14.6	51.3 ± 15.5	53.3 ± 16.0
Sex (male)	1,769,429 (63.6)	1,868,857 (58.2)	905,309 (53.9)	238,342 (50.7)	28,387 (48.2)
FBG (mg/dL)	95.4 ± 17.0	96.7 ± 21.2	98.7 ± 25.5	101.0 ± 30.0	104.0 ± 35.2
TC (mg/dL)	196.3 ± 33.2	195.6 ± 35.8	195.2 ± 38.9	194.9 ± 42.4	193.8 ± 45.5
HDL cholesterol (mg/dL)	54.7 ± 19.0	55.1 ± 19.9	55.4 ± 20.6	55.6 ± 21.9	55.3 ± 21.9
LDL cholesterol (mg/dL)	116.6 ± 44.5	115.1 ± 46.1	114.0 ± 47.7	112.8 ± 48.8	111.3 ± 49.3
Triglyceride (mg/dL)	110 (75–163)	110 (76–164)	112 (77–166)	114 (79–170)	117 (80–174)
eGFR (mL/min/1.73 m^2^)	86.5 ± 42.6	87.0 ± 40.1	87.2 ± 39.2	87.1 ± 39.3	86.7 ± 39.0
eGFR < 60 mL/min/1.73m^2^	167,159 (6.0)	201,998(6.3)	121,175 (7.2)	40,717 (8.7)	6287 (10.7)
Proteinuria (yes) ^a^	49,727 (1.8)	68,587(2.1)	43,510 (2.6)	15,293 (3.3)	2442 (4.2)
Systolic BP (mmHg)	122.5 ± 13.0	122.4 ± 14.6	122.6 ± 15.9	122.8 ± 17.3	123.0 ± 18.8
Diastolic BP (mmHg)	76.6 ± 9.3	76.4 ± 9.8	76.3 ± 10.2	76.2 ± 10.7	76.1 ± 11.3
BMI (kg/m^2^)	23.7 ± 3.0	23.8 ± 3.1	23.8 ± 3.3	23.8 ± 3.4	23.7 ± 3.5
Waist circumferences (cm)	80.5 ± 8.7	80.5 ± 8.9	80.7 ± 9.1	80.9 ± 9.2	81.0 ± 9.4
Variability					
VIM of FBG (%)	7.1 ± 3.1	9.9 ± 5.7	12.5 ± 6.6	15.4 ± 6.6	18.6 ± 5.4
VIM of TC (%)	13. 8 ± 5.7	18.7 ± 10.7	24.7 ± 13.0	30.9 ± 13.2	36.6 ± 11.5
VIM of systolic BP (%)	6.9 ± 2.9	9.3 ± 4. 9	11.4 ± 5.5	13.6 ± 5.4	16.4 ± 4.0
VIM of diastolic BP (%)	5.85 ± 3.0	6.6 ± 3.6	7.4 ± 3.9	8.3 ± 4.1	9.4 ± 4.1
VIM of BMI (%)	0.5 ± 0.2	0.7 ± 0.5	1.0 ± 0.6	1.2 ± 0.7	1.5 ± 0.7
Current smoker (yes)	724,804 (26.1)	815,211 (25.4)	406,455 (24.2)	108,387 (23.0)	12,866 (21.9)
Heavy alcohol drinker (yes)	218,920 (7.9)	244,023 (7.6)	123,648 (7.4)	34,132 (7.3)	4116 (7.0)
Regular Exercise	570,589 (20.5)	635,744 (19.8)	318,960 (19.0)	84,638 (18.0)	9902 (16.8)
Income (lower 25%)	396,775 (14.3)	527,799 (16.5)	307,135 (18.3)	91,791 (19.5)	12,024 (20.4)
Diabetes mellitus	138,223 (5.0)	261,325 (8.1)	205,021 (12.2)	82,009 (17.4)	14,272 (24.2)
Hypertension	594,422 (21.4)	835,561 (26.0)	525,243 (31.3)	173,001 (36.8)	25,115 (42.6)
Dyslipidemia	314,731 (11.3)	502,007 (15.6)	343,067 (20.4)	117,774 (25.0)	17,128 (29.1)

Data are expressed as the means ± SD, median (25–75%), or *n* (%). *p*-values for the trend were <0.0001 for all variables because of the large size of the study population. Body mass index (BMI); blood pressure (BP); estimated glomerular filtration rate (eGFR); fasting blood glucose (FBG); high-density lipoprotein (HDL); low-density lipoprotein (LDL); total cholesterol (TC); variability independent of the mean (VIM). ^a^ Proteinuria was defined as having urinary protein ≥1+ on dipstick testing in fasting morning urine.

**Table 2:** Baseline characteristics of subjects according to the incident end-stage renal disease.

	No ESRD (*n* = 8,185,535)	ESRD (*n* = 13,600)
Age (years)	48.2 ± 13.7	60.9 ± 13.0
Sex (male)	4,800,955 (58.7)	9369 (68.9)
FBG (mg/dL)	96.9 ± 21.6	118.8 ± 50.9
TC (mg/dL)	195.7 ± 36.1	192.6 ± 44.9
HDL cholesterol (mg/dL)	55.1 ± 19.9	50.2 ± 23.8
LDL cholesterol (mg/dL)	115.2 ± 46.1	111.4 ± 48.2
Triglyceride (mg/dL)	111 (76–165)	136 (96–199)
eGFR (mL/min/1.73 m^2^)	86.9 ± 40.7	54.7 ± 34.9
eGFR <60mL/min/1.73m^2^	529,140 (6.5)	8196 (60.3)
Proteinuria ^a^	173,792 (2.1)	5767 (42.4)
Systolic BP (mmHg)	122.5 ± 14.5	132.3 ± 17.8
Diastolic BP (mmHg)	76.4 ± 9.8	79.5 ± 11.0
BMI (kg/m^2^)	23.7 ± 3.1	24.1 ± 3.2
Waist circumferences (cm)	80.6 ± 8.9	84.2 ± 8.9
Variability		
VIM of FBG (%)	9.9 ± 5.8	12.2 ± 8.0
VIM of TC (%)	19.1 ± 11.3	26.3 ± 15. 8
VIM of systolic BP (%)	9.2 ± 5.0	10.9 ± 6.0
VIM of diastolic BP (%)	6.6 ± 3.6	7.7 ± 4.3
VIM of BMI (%)	0.73 ± 0.51	0.86 ± 0.62
Current smoker (yes)	2,064,667 (25.2)	3056 (22.5)
Heavy alcohol drinker (yes)	624,100 (7.6)	739 (5.4)
Regular Exercise	1,617,018 (19.8)	2815 (20.7)
Income (lower 25%)	1,332,692 (16.3)	2832 (20.8)
Diabetes mellitus	694,862 (8.5)	5988 (44.0)
Hypertension	2,142,688 (26.2)	10,654 (78.3)
Dyslipidemia	1,289,041 (15.8)	5666 (41.7)

Data are expressed as the means ± SD, median (25–75%), or *n* (%). *p*-values were <0.0001 for all variables because of the large size of the study population. Body mass index (BMI); blood pressure (BP); estimated glomerular filtration rate (eGFR); end-stage renal disease (ESRD); fasting blood glucose (FBG); high-density lipoprotein (HDL); low-density lipoprotein (LDL); total cholesterol (TC); variability independent of the mean (VIM). ^a^ Proteinuria was defined as having urinary protein ≥1+ on dipstick testing in fasting morning urine.

**Table 3:** Hazard ratios and 95% confidence intervals of end-stage renal disease by quartiles of metabolic parameters variability.

	Events (n)	Follow-Up Duration (Person-Year)	Incidence Rate (per 1000 Person-Years)	Model 1	Model 2	Model 3
Glucose variability (VIM of FBG)						
Q1	2771	14,077,785	0.20	1 (ref.)	1 (ref.)	1 (ref.)
Q2	2702	14,204,338	0.19	1.02 (0.96,1.07)	1.01 (0.96,1.06)	1.01 (0.96,1.06)
Q3	3090	14,240,501	0.22	1.16 (1.11,1.23)	1.13 (1.07,1.19)	1.16 (1.11,1.22)
Q4	5037	14,201,590	0.35	1.73 (1.65,1.81)	1.46 (1.40,1.53)	1.47 (1.40,1.54)
*p* for trend				<0.0001	<0.0001	<0.0001
Cholesterol variability (VIM of TC)						
Q1	2091	14,144,226	0.15	1 (ref.)	1 (ref.)	1 (ref.)
Q2	2276	14,264,721	0.16	1.12 (1.05,1.19)	1.11 (1.04,1.17)	1.06 (1.00,1.13)
Q3	2923	14,240,930	0.21	1.39 (1.31,1.47)	1.35 (1.27,1.43)	1.31 (1.23,1.38)
Q4	6310	14,074,337	0.45	2.52 (2.40,2.65)	2.27 (2.16,2.39)	2.08 (1.97,2.18)
*p* for trend				<0.0001	<0.0001	<0.0001
Blood pressure variability (VIM of systolic BP)						
Q1	2794	14,348,779	0.19	1 (ref.)	1 (ref.)	1 (ref.)
Q2	2591	14,032,640	0.18	1.02 (0.97,1.08)	1.04 (0.98,1.10)	0.98 (0.93,1.04)
Q3	3165	14,160,292	0.22	1.13 (1.07,1.19)	1.12 (1.07,1.18)	1.08 (1.03,1.14)
Q4	5050	14,182,504	0.36	1.53 (1.46,1.61)	1.52 (1.45,1.59)	1.46 (1.39,1.53)
*p* for trend				<0.0001	<0.0001	<0.0001
BMI variability (VIM of BMI)						
Q1	2909	14,166,998	0.21	1 (ref.)	1 (ref.)	1 (ref.)
Q2	2753	14,261,067	0.19	1.00 (0.94,1.05)	0.99 (0.94,1.04)	0.98 (0.93,1.04)
Q3	3319	14,223,477	0.23	1.21 (1.15,1.27)	1.20 (1.14,1.26)	1.20 (1.14,1.26)
Q4	4619	14,072,671	0.33	1.65 (1.57,1.72)	1.58 (1.51,1.66)	1.56 (1.49,1.64)
*p* for trend				<0.0001	<0.0001	<0.0001

Model 1: Adjusted for age, sex, alcohol drinking, smoking, regular exercise, and income status. Model 2: Adjusted for Model 1 plus baseline fasting glucose levels, total cholesterol, systolic blood pressure, and body mass index. Model 3; adjusted for Model 3 plus glomerular filtration rate and proteinuria. Body mass index (BMI); blood pressure (BP); fasting blood glucose (FBG); total cholesterol (TC); variability independent of the mean (VIM).

**Table 4:** Hazard ratios and 95% confidence intervals of end-stage renal disease by the number of high variability in the metabolic parameters.

	Events (*n*)	Follow-Up Duration (Person-Years)	Incidence Rate (Per 1000 Person-Years)		Model 1	Model 2	Model 3
**VIM**							
0	2336	19,352,484	0.12		1 (ref.)	1 (ref.)	1 (ref.)
1	4540	22,215,048	0.20		1.57 (1.49,1.65)	1.48 (1.41,1.56)	1.54 (1.47,1.62)
2	4157	11,550,224	0.36		2.48 (2.36,2.61)	2.21 (2.10,2.32)	2.25 (2.14,2.37)
3	2106	3,210,226	0.66		4.02 (3.79,4.27)	3.36 (3.16,3.57)	3.17 (2.99,3.37)
4	461	396,231	1.16		6.33 (5.73,7.00)	4.93 (4.45,5.45)	4.12 (3.72,4.57)
*p* for trend					<0.0001	<0.0001	<0.0001
**CV**							
0	1884	19,622,314	0.10		1 (ref.)	1 (ref.)	1 (ref.)
1	4120	21,937,268	0.19		1.74 (1.65,1.84)	1.57 (1.49,1.66)	1.65 (1.56,1.74)
2	4335	11,471,033	0.38		3.03 (2.87,3.20)	2.46 (2.32,2.60)	2.50 (2.37,2.65)
3	2629	3,271,586	0.80		5.54 (5.22,5.88)	4.03 (3.78,4.28)	3.74 (3.51,3.98)
4	632	422,013	1.50		8.91 (8.13,9.76)	5.85 (5.33,6.42)	4.95 (4.51,5.44)
*p* for trend					<0.0001	<0.0001	<0.0001
**ARV**							
0	1698	19,749,696	0.09	1 (ref.)		1 (ref.)	1 (ref.)
1	3884	21,390,895	0.18	1.75 (1.66,1.86)		1.57 (1.48,1.66)	1.66 (1.57,1.76)
2	4503	11,527,893	0.39	3.14 (2.97,3.33)		2.50 (2.36,2.65)	2.57 (2.42,2.72)
3	2757	3,546,361	0.78	5.32 (5.00,5.66)		3.78 (3.54,4.02)	3.56 (3.34,3.80)
4	758	509,369	1.49	8.97 (8.22,9.78)		5.69 (5.21,6.22)	4.82 (4.41,5.28)
*p* for trend				<0.0001		<0.0001	<0.0001

Model 1: Adjusted for age, sex, alcohol drinking, smoking, regular exercise, and income status. Model 2: Adjusted for Model 1 plus baseline fasting glucose levels, total cholesterol, systolic blood pressure, and body mass index. Model 3: Adjusted for Model 3 plus glomerular filtration rate and proteinuria. average real variability (ARV); coefficient of variation (CV); variability independent of the mean (VIM).

**Table 5:** Hazard ratios and 95% confidence intervals of end-stage renal disease by the number of high variabilities in the metabolic parameters: Sensitivity analysis excluding subjects having diabetes mellitus, hypertension, or dyslipidemia.

	Events (*n*)	Follow-Up Duration (Person-Years)	Incidence Rate (Per 1000 Person-Years)	Model 1	Model 2	Model 3
**VIM**						
0	517	13,581,340	0.04	1 (ref.)	1(ref.)	1 (ref.)
1	667	14,105,586	0.05	1.22 (1.09,1.37)	1.22 (1.09,1.37)	1.24 (1.10,1.39)
2	393	6,511,970	0.06	1.48 (1.30,1.69)	1.47 (1.29,1.68)	1.50 (1.32,1.72)
3	166	1,584,979	0.10	2.40 (2.01,2.86)	2.38 (1.99,2.84)	2.40 (2.01,2.87)
4	25	168,698	0.15	3.15 (2.11,4.72)	3.09 (2.07,4.63)	2.98 (1.99,4.46)
*p* for trend				<0.0001	<0.0001	<0.0001
**CV**						
0	542	14,500,895	0.04	1 (ref.)	1 (ref.)	1 (ref.)
1	662	13,925,803	0.05	1.22 (1.09,1.36)	1.21 (1.08,1.36)	1.23 (1.10,1.38)
2	378	6,023,625	0.06	1.48 (1.30,1.70)	1.47 (1.29,1.68)	1.50 (1.31,1.71)
3	159	1,368,232	0.12	2.45 (2.05,2.93)	2.41 (2.02,2.88)	2.45 (2.05,2.93)
4	27	134,019	0.20	3.69 (2.50,5.44)	3.59 (2.43,5.29)	3.42 (2.32,5.05)
*p* for trend				<0.0001	<0.0001	<0.0001
**ARV**						
0	548	15,328,355	0.04	1 (ref.)	1(ref.)	1 (ref.)
1	652	13,587,492	0.05	1.20 (1.07,1.35)	1.21 (1.08,1.36)	1.23 (1.10,1.38)
2	425	5,648,100	0.08	1.65 (1.45,1.88)	1.68 (1.48,1.91)	1.72 (1.51,1.95)
3	117	1,264,835	0.09	1.74 (1.42,2.13)	1.78 (1.45,2.18)	1.80 (1.47,2.21)
4	26	123,791	0.21	3.34 (2.25,4.95)	3.42 (2.30,5.08)	3.32 (2.23,4.93)
*p* for trend				<0.0001	<0.0001	<0.0001

Model 1: Adjusted for age, sex, alcohol drinking, smoking, regular exercise, and income status. Model 2: Adjusted for Model 1 plus baseline fasting glucose levels, total cholesterol, systolic blood pressure, and body mass index. Model 3: Adjusted for Model 3 plus glomerular filtration rate and proteinuria. Average real variability (ARV); coefficient of variation (CV); variability independent of the mean (VIM).

## Supplementary Materials

The following are available online at https://www.mdpi.com/2077-0383/8/5/755/s1, Figure S1. Flow chart of the study population, Figure S2. Schematic description of the study period, Table S1. Hazard ratios and 95% confidence intervals of ESRD by the number of high variability (measured by VIM) in the metabolic parameters: Sensitivity analysis excluding subjects with the occurrence of end points within 2 years of follow-up, Table S2. Hazard ratios and 95% confidence intervals of ESRD by the number of high variability (measured by VIM) in the metabolic parameters: Sensitivity analysis confined to subjects with yearly measurements of metabolic parameters (n = 1,460,693), Table S3. Combined effects of the variability in FBG and SBP, SBP and TC, and FBG and TC on the risk of ESRD.
