# Supplementary material for: Effects of Variability in Blood Pressure, Glucose, and Cholesterol Concentrations, and Body Mass Index on End-Stage Renal Disease in the General Population of Korea
Source: J Clin Med. 2019 May 27;8(5):755. doi: 10.3390/jcm8050755 (PMC6571839; doi:10.3390/jcm8050755)

Table S1. Hazard ratios and 95% confidence intervals of ESRD by the number of high variability (measured by VIM) in the metabolic parameters: Sensitivity analysis excluding subjects with the occurrence of end points within 2 years of follow-up

	Events (*n*)	Follow-up duration (person-year)	Incidence rate (per 1000 person-years)	Model 1	Model 2	Model 3
Glucose variability (VIM of FBG)						
Q1	2267	9984954	0.23	1(ref.)	1(ref.)	1(ref.)
Q2	2193	10109244	0.23	1.01(0.95,1.07)	1.00(0.94,1.06)	1.00(0.94,1.06)
Q3	2534	10147136	0.25	1.16(1.10,1.23)	1.13(1.07,1.19)	1.15(1.09,1.22)
Q4	3939	10111275	0.39	1.64(1.56,1.73)	1.37(1.30,1.44)	1.37(1.30,1.45)
P for trend				<0.0001	<0.0001	<0.0001
Cholesterol variability (VIM of TC)						
Q1	1753	10050194	0.17	1(ref.)	1(ref.)	1(ref.)
Q2	1863	10170529	0.18	1.09(1.02,1.17)	1.08(1.01,1.15)	1.04(0.98,1.11)
Q3	2399	10147207	0.24	1.36(1.28,1.44)	1.32(1.24,1.40)	1.28(1.20,1.36)
Q4	4918	9984679	0.49	2.34(2.21,2.47)	2.10(1.99,2.22)	1.93(1.83,2.04)
P for trend				<0.0001	<0.0001	<0.0001
Blood pressure variability (VIM of systolic BP)						
Q1	2277	10194835	0.22	1(ref.)	1(ref.)	1(ref.)
Q2	2140	9998601	0.21	1.04(0.98,1.10)	1.05(0.99,1.12)	1.00(0.95,1.06)
Q3	2578	10094368	0.26	1.13(1.06,1.19)	1.12(1.06,1.19)	1.09(1.03,1.15)
Q4	3938	10064805	0.39	1.46(1.39,1.54)	1.46(1.38,1.53)	1.40(1.33,1.47)
P for trend				<0.0001	<0.0001	<0.0001
BMI variability (VIM of BMI)						
Q1	2428	10072237	0.24	1(ref.)	1(ref.)	1(ref.)
Q2	2258	10166942	0.22	0.98(0.92,1.03)	0.97(0.92,1.03)	0.97(0.92,1.03)
Q3	2682	10130064	0.26	1.17(1.11,1.24)	1.16(1.10,1.22)	1.16(1.10,1.23)
Q4	3565	9983367	0.36	1.52(1.45,1.61)	1.46(1.39,1.54)	1.46(1.38,1.54)
P for trend				<0.0001	<0.0001	<0.0001
Number of high-variability parameters						
0	1997	13793351	0.14	1(ref.)	1(ref.)	1(ref.)

1	3766	15805834	0.24	1.51(1.43,1.60)	1.43(1.35,1.51)	1.47(1.39,1.55)
2	3255	8201229	0.40	2.26(2.13,2.39)	1.99(1.88,2.11)	2.02(1.91,2.14)
3	1576	2272946	0.69	3.50(3.27,3.74)	2.89(2.70,3.09)	2.72(2.54,2.92)
4	339	279248	1.21	5.42(4.82,6.08)	4.15(3.69,4.66)	3.54(3.15,3.98)
P for trend				<0.0001	<0.0001	<0.0001

Model 1: Adjusted for age, sex, alcohol drinking, smoking, regular exercise and income status

Model 2: Adjusted for Model 1 plus baseline fasting glucose levels, total cholesterol, systolic blood pressure and body mass index

Model 3; Adjusted for Model 3 plus glomerular filtration rate and proteinuria.

VIM, variability independent of the mean; FBG, fasting blood glucose; TC, total cholesterol; BP, blood pressure; BMI, body mass index

Table S2. Hazard ratios and 95% confidence intervals of ESRD by the number of high variability (measured by VIM) in the metabolic parameters: Sensitivity analysis confined to subjects with yearly measurements of metabolic parameters (*n* = 1,460,693)

	Events (*n*)	Follow-up duration (person-year)	Incidence rate (per 1000 person-years)	Model 1	Model 2	Model 3
Glucose variability (VIM of FBG)						
Q1	176	1818081	0.10	1(ref.)	1(ref.)	1(ref.)
Q2	304	2883331	0.11	1.13(0.94,1.36)	1.14(0.94,1.37)	1.30(1.08,1.56)
Q3	427	3137428	0.14	1.45(1.22,1.73)	1.43(1.20,1.71)	1.80(1.51,2.15)
Q4	544	2822486	0.19	1.91(1.61,2.27)	1.60(1.35,1.90)	2.03(1.71,2.42)
P for trend				<0.0001	<0.0001	<0.0001
Cholesterol variability (VIM of TC)						
Q1	143	2099771	0.07	1(ref.)	1(ref.)	1(ref.)
Q2	247	3362184	0.07	1.09(0.89,1.34)	1.07(0.87,1.32)	1.07(0.87,1.32)
Q3	416	3187099	0.13	1.93(1.60,2.34)	1.85(1.53,2.24)	1.85(1.53,2.24)
Q4	645	2012272	0.32	4.39(3.66,5.27)	3.88(3.23,4.65)	3.66(3.05,4.40)
P for trend				<0.0001	<0.0001	<0.0001
Blood pressure variability (VIM of systolic BP)						
Q1	214	2153873	0.10	1(ref.)	1(ref.)	1(ref.)
Q2	325	3102086	0.11	1.03(0.87,1.23)	1.03(0.87,1.22)	1.00(0.84,1.19)
Q3	449	3376400	0.13	1.30(1.10,1.53)	1.31(1.11,1.54)	1.30(1.10,1.53)
Q4	463	2028967	0.23	2.10(1.78,2.47)	2.04(1.74,2.41)	1.96(1.66,2.31)
P for trend				<0.0001	<0.0001	<0.0001
BMI variability (VIM of BMI)						
Q1	289	2155746	0.13	1(ref.)	1(ref.)	1(ref.)
Q2	364	3260048	0.11	0.86(0.74,1.01)	0.86(0.73,1.00)	0.87(0.75,1.02)
Q3	445	3025043	0.15	1.23(1.06,1.43)	1.20(1.04,1.40)	1.23(1.06,1.43)
Q4	353	2220489	0.16	1.57(1.35,1.84)	1.43(1.22,1.67)	1.47(1.25,1.71)
P for trend				<0.0001	<0.0001	<0.0001
Number of high-variability parameters						
0	322	4339046	0.07	1(ref.)	1(ref.)	1(ref.)
1	500	4076469	0.12	1.67(1.45,1.92)	1.56(1.35,1.79)	1.67(1.45,1.92)
2	417	1775308	0.23	3.19(2.75,3.69)	2.75(2.37,3.18)	2.89(2.49,3.35)

3	177	424883	0.42	5.64(4.69,6.78)	4.40(3.65,5.31)	4.42(3.65,5.34)
4	35	45619	0.77	10.16(7.16,14.43)	7.44(5.23,10.58)	7.30(5.13,10.40)
P for trend				<0.0001	<0.0001	<0.0001

Model 1: Adjusted for age, sex, alcohol drinking, smoking, regular exercise and income status

Model 2: Adjusted for Model 1 plus baseline fasting glucose levels, total cholesterol, systolic blood pressure and body mass index

Model 3; Adjusted for Model 3 plus glomerular filtration rate and proteinuria.

VIM, variability independent of the mean; FBG, fasting blood glucose; TC, total cholesterol; BP, blood pressure; BMI, body mass index

Table S3. Combined effects of the variability in FBG and SBP, SBP and TC, and FBG and TC on the risk of ESRD.

	Events (*n*)	Incidence Rate (Per 1000 Person-Years)	Model 1	Model 2	Model 3
Glucose variability (VIM of FBG)					
Q1-3 (low variability)	8563	0.20	1 (ref.)	1 (ref.)	1 (ref.)
Q4 (high variability)	5037	0.35	1.63(1.57,1.69)	1.40(1.35,1.45)	1.39(1.34,1.44)
Blood pressure variability (VIM of SBP)					
Q1-3 (low variability)	8550	0.20	1 (ref.)	1 (ref.)	1 (ref.)
Q4 (high variability)	5050	0.36	1.46(1.41,1.51)	1.44(1.39,1.49)	1.43(1.38,1.48)
Cholesterol variability (VIM of TC)					
Q1-3 (low variability)	7290	0.17	1 (Ref.)	1 (Ref.)	1 (Ref.)
Q4 (high variability)	6310	0.45	2.15(2.08,2.23)	1.97(1.90,2.04)	1.84(1.78,1.91)
Combination of FBG variability and SBP variability					
Low variability of FBG/low variability of SBP	5491	0.17	1 (ref.)	1 (ref.)	1 (ref.)
Low variability of FBG /High variability of SBP	3072	0.29	1.428(1.366,1.493)	1.427(1.365,1.492)	1.441(1.378,1.507)
High variability of FBG/ low variability of SBP	3059	0.29	1.602(1.532,1.674)	1.386(1.325,1.449)	1.403(1.341,1.467)
High variability of FBG/High variability of SBP	1978	0.53	2.345(2.226,2.469)	1.983(1.882,2.09)	1.918(1.819,2.021)
Combination of SBP variability and TC variability					
Low variability of SBP/low variability of TC	4744	0.15	1 (ref.)	1 (ref.)	1 (ref.)
Low variability of SBP /High variability of TC	3806	0.37	2.12(2.03,2.22)	1.94(1.85,2.02)	1.87(1.79,1.96)
High variability of SBP/ low variability of TC	2546	0.25	1.42(1.35,1.49)	1.40(1.34,1.47)	1.45(1.38,1.52)
High variability of SBP/High variability of TC	2504	0.64	3.05(2.91,3.21)	2.78(2.64,2.92)	2.52(2.40,2.65)

Combination of FBG variability and TC variability					
Low variability of FBG/low variability of TC	4856	0.15	1 (ref.)	1 (ref.)	1 (ref.)
Low variability of FBG/High variability of TC	3707	0.37	2.05(1.96,2.14)	1.90(1.82,1.98)	1.83(1.75,1.91)
High variability of FBG/ low variability of TC	2434	0.24	1.51(1.44,1.58)	1.32(1.25,1.38)	1.36(1.29,1.43)
High variability of FBG/High variability of TC	2603	0.64	3.30(3.15,3.47)	2.66(2.53,2.79)	2.43(2.31,2.55)

Model 1: Adjusted for age, sex, alcohol drinking, smoking, regular exercise and income status
Model 2: Adjusted for Model 1 plus baseline fasting glucose levels, total cholesterol, systolic blood pressure and body mass index
Model 3; Adjusted for Model 3 plus glomerular filtration rate and proteinuria.
FBG, fasting blood glucose; SBP, systolic blood pressure; TC, total cholesterol; VIM, variability independent of the mean

Figure S1. Flow chart of the study population

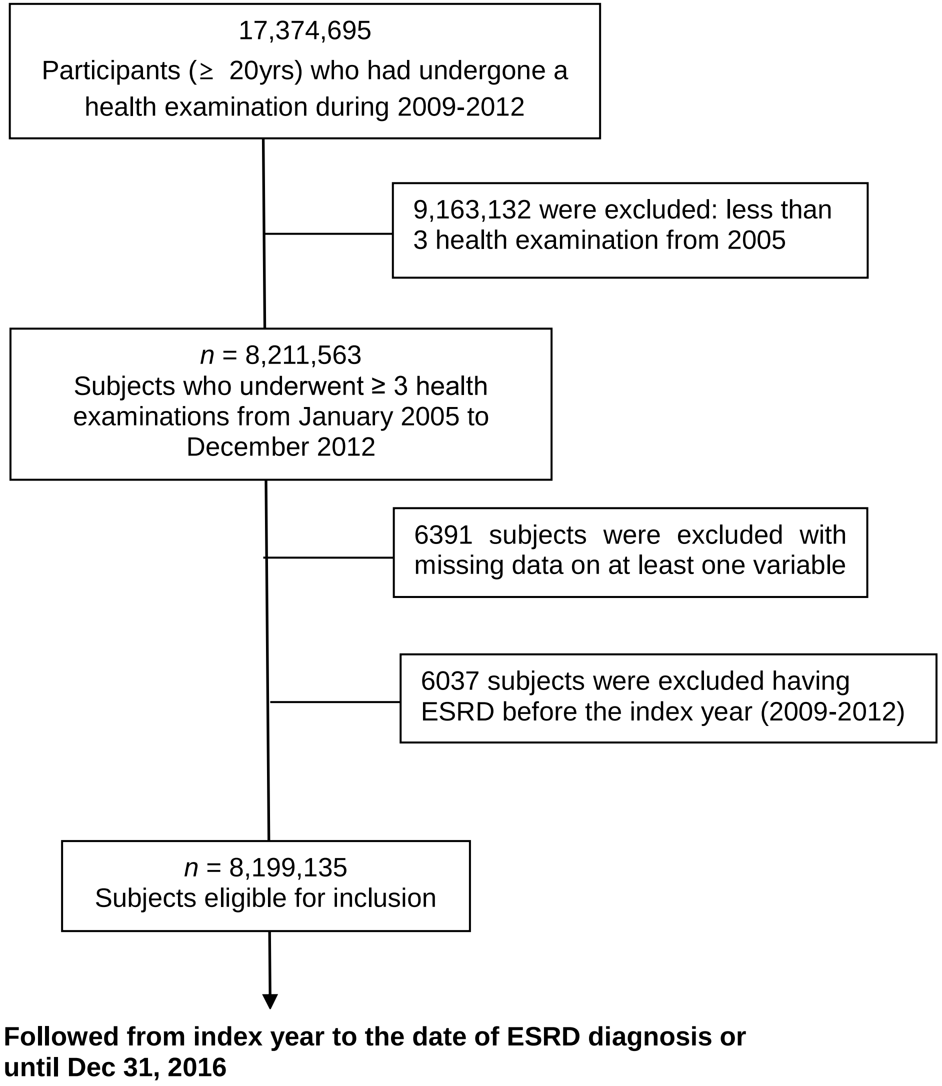

Figure S2. Schematic description of the study period.

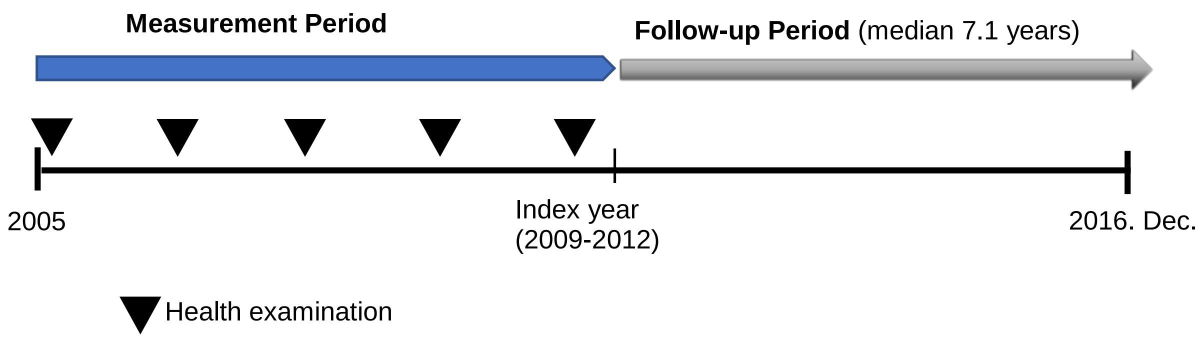

Supplement: Supplementary file 1 [file jcm-08-00755-s001.pdf]
